# Prevalence of hepatitis C virus among street children in Iran

**DOI:** 10.1186/s40249-018-0469-5

**Published:** 2018-10-01

**Authors:** Masoud Behzadifar, Hasan Abolghasem Gorji, Aziz Rezapour, Nicola Luigi Bragazzi

**Affiliations:** 1 Department of Health Services Management, School of Health Management and Information Sciences, Iran University of Medical Sciences, Tehran, Iran; 2 Health Management and Economics Research Center, Iran University of Medical Sciences, Tehran, Iran; 3 School of Public Health, Department of Health Sciences (DISSAL), University of Genoa, Genoa, Italy

**Keywords:** Systematic review, Meta-analysis, Hepatitis C virus, Street children, Iran

## Abstract

**Background:**

Street children are forced to spend a lot of time away from their homes and some of them do not have homes at all, due to economic and family problems, which makes them exposed to many health problems, such as the hepatitis C virus (HCV) infection. Iran, like many other countries in the world, experiences the burden of street children, however, the rate of HCV among street children is virtually unknown. This study aimed to determine the prevalence of HCV among street children in Iran.

**Main body:**

This systematic review and meta-analysis was performed according to the Preferred Reporting Items for Systematic Reviews and Meta-Analyses guidelines. The study protocol of this review was registered in PROSPERO under identification term CRD42018082336. A comprehensive literature search was conducted to identify published studies reporting on the prevalence of HCV among street children in Iran. Several international scholarly databases, including Web of Science, PubMed®/MEDLINE®, Embase, Scopus®, Google Scholar and Directory of Open Access Journals, as well as Iranian databases such as MagIran and Barakathns were searched. Studies published between 1988 and December 2017 with any of the following keywords were selected: (street OR homeless OR labour) AND (children OR child OR infant) AND (hepatitis C OR hepatitis C virus OR HCV OR viral hepatitis OR hepatitis OR hepacivirus) AND Iran. Moreover, a grey literature search was performed in order to obtain other potentially relevant studies. The search was carried out without any language restrictions. Four studies, surveying a total of 1691 street children, conducted between 2006 and 2017 were found to be eligible for inclusion in the review and therefore analysed. Three studies were conducted in Tehran and one in Isfahan. The prevalence of HCV among street children in Iran was found to be high, at 2.4% (95% *CI*: 1.8–3.3).

**Conclusions:**

Since the prevalence of HCV among street children in Iran is quite high, health decision- and policy-makers should pay more attention to street children and widen support channels, both social and economic. Further studies should be conducted among street children in different cities of Iran to add to the knowledge base of HCV among street children in the country. The health system should provide facilities for street children to be screened to quickly diagnose illnesses and prevent them from developing complications.

**Electronic supplementary material:**

The online version of this article (10.1186/s40249-018-0469-5) contains supplementary material, which is available to authorized users.

## Multilingual abstracts

Please see Additional file 1 for translations of the abstract into the five official working languages of the United Nation.

## Background

A street or labour child is “any girl or boy who has not reached adulthood, for whom the street (in the broadest sense of the word, including unoccupied dwellings, wasteland, etc.) has become her or his habitual abode and/or sources of livelihood, and who is inadequately protected, supervised or directed by responsible adults” [1]. Street children are exposed to many health problems [2] due to their poor living conditions and are at risk of developing serious communicable illnesses such as the human immunodeficiency virus (HIV(, viral hepatitis such as hepatitis B virus (HBV) and hepatitis C virus (HCV), sexually transmitted diseases (STDs) and other infections [3, 4].

Among these diseases, HCV is one of the main public health concerns worldwide, imposing a dramatic economic burden for healthcare systems [5]. The World Health Organization estimates that around 71 million people in the world are infected with chronic hepatitis C infection [6]. Children are particularly at risk and, unfortunately, there is scarce information regarding the prevalence of HCV in this age group. HCV in children is often asymptomatic, and a screening policy should be considered as a beneficial strategy for this group [7, 8].

In Iran, HCV is considered a public health challenge, with the Ministry of Health trying to combat the disease by implementing various programmes. According to a recent meta-analysis published in 2018, the prevalence of HCV in the general population of Iran is about 0.3% and among high-risk populations, it is 32.1% [9]. The prevalence of HCV among street children in Iran is virtually unknown.

Since knowledge is crucial for making appropriate decisions about people’s health, this gap in knowledge has prevented health policy- and decision-makers from having a clear picture of the situation pertaining to street children. Moreover, in Iran few studies have been conducted on the prevalence of HCV among street children. Given the clinical and epidemiological relevance of this problem, this study aimed to systematically determine the prevalence of HCV among street children in Iran by synthesising existing literature utilising a rigorous, transparent and reproducible approach. A systematic review and meta-analysis is the best way to collate evidence, and pooling together different studies can increase their statistical power and significance.

## Methods

This study adhered to the Preferred Reporting Items for Systematic Reviews and Meta-Analyses (PRISMA) guidelines [10]. The study protocol of this review was registered in PROSPERO under the identification term CRD42018082336.

### Literature search

Several international scholarly databases were searched, including Web of Science, PubMed®/MEDLINE®, Embase, Scopus®, Google Scholar and Directory of Open Access Journals, as well as Iranian databases such as MagIran and Barakathns.

Articles published between 1988 (the year in which HCV was described and confirmed) and December 2017 featuring the following keywords were selected: (street OR homeless OR labour) AND (children OR child OR infant) AND (hepatitis C OR hepatitis C virus OR HCV OR viral hepatitis OR hepatitis OR hepacivirus) AND Iran. This search strategy was adopted without any language restrictions. Reference lists of each study were also reviewed and hand-searched to increase the chance of finding relevant studies.

### Outcome(s)

The outcomes of the present study are the prevalence of HCV (primary outcome) and the risk factors for HCV (secondary outcome) in street children in Iran. If enough data (in terms of socio-demographic variables and other parameters related to risk factors) are provided, it was decided that odds ratios (*OR*s) will be calculated.

### Inclusion and exclusion criteria

Studies were included if: 1) the investigations were conducted as observational studies (cross-sectional, cohort, case-control) reporting the prevalence of HCV in street children in Iran; 2) the prevalence could be computed based on available data, and 3) the studies used valid screening tests for the diagnosis of HCV. Studies were excluded if: 1) the investigations reported unclear or insufficient quantitative data, and 2) the studies provided overlapping data.

### Data extraction

Two authors independently extracted data from the studies. Data extracted included the name of the first author, the year of publication, the location, sample size, age of subjects, the test used to screen HCV and the number of positive patients. Any disagreement was resolved by a discussion.

### Evaluating the quality of the studies

To evaluate the methodological quality of the studies, the STrengthening the Reporting of OBservational studies in Epidemiology (STROBE) checklist was used [11]. Based on this checklist, studies that received 1–7 points were deemed of poor quality, 8–15 were considered of medium quality and 16–22 points were judged to be of high quality.

### Statistical analysis

To estimate the prevalence of HCV among Iranian street children, the DerSimonian and Laird fixed-effects model with 95% confidence intervals (*CI*s) [12] was used. An initial, exploratory analysis was performed and as the heterogeneity among studies was found to be less than 50%, a fixed-effects model was then used.

To calculate the prevalence of HCV, the number of people who tested positive was determined and then this number was divided by the total number of study participants, as shown below:

$$ p=\frac{number\ of\  HCV\  patients+}{number\ of\ participants\ } $$

$$ q=1-p $$

$$ standard\ error=\sqrt{\frac{p\bullet \left(1-q\right)}{n}} $$

The heterogeneity of the studies was evaluated using the *I*^2^ test [13]. As the number of studies retained in the current systematic review and meta-analysis was less than ten, it was not possible to estimate the publication bias [14]. The stability of the results was investigated by sensitivity analysis, evaluating the impact of removing each individual study.

Comprehensive meta-analysis (CMA) version 2 software (Biostat, Englewood, NJ, USA) was used to analyse the data. Figures with *P*-values of less than 0.05 were considered to be statistically significant.

## Results

**Fig. 1:**
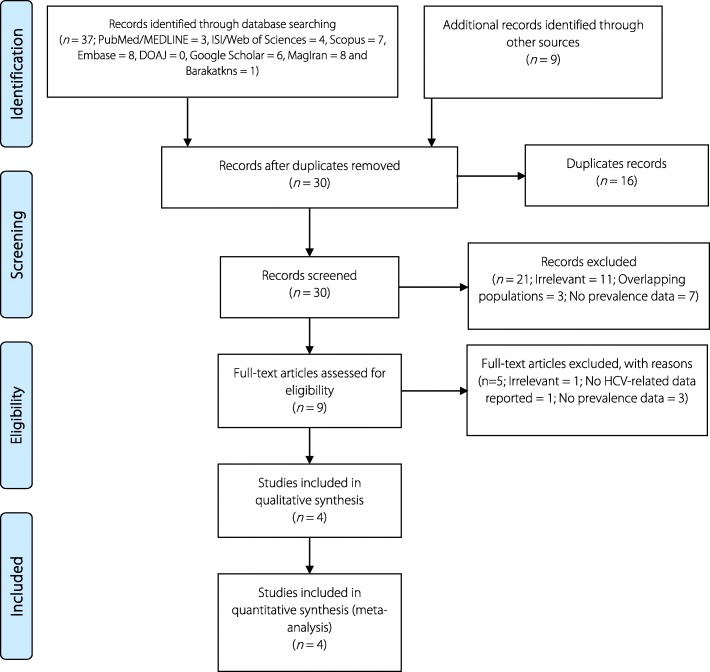
The process of searching for and selecting the studies

In the initial search, 46 studies were found and after the removal of duplicates, 30 studies were reviewed by two authors. After reviewing the title and abstract, four studies were found to be eligible for inclusion in the current review and were therefore analysed [15–18]. Figure 1 shows the process of retrieval and selection of the studies.

The four studies, surveying a total of 1691 street children, were conducted between 2006 and 2017. All investigations were cross-sectional studies. Three studies were conducted in Tehran and one study in Isfahan. The characteristics of the studies are summarised in Table 1.

**Table 1:** The characteristics of studies

First author	Year	City	Sample size	Number of participants infected with HCV	Gender of infected with HCV	Age (Year±SD)	Odds ratio of risk factor reported	Study design	Test
Vahdani	2006	Tehran	102	0	0	10.1± 3	Not reported	Cross-sectional	ELISA
Fallah	2008	Tehran	203	7	Boy=7,Girl=0	NA	Not reported	Cross-sectional	ELISA
Ataei	2010	Isfahan	386	4	Boy=4,Girl=0	12.62±3.23	Not reported	Cross-sectional	ELISA
Foroughi	2017	Tehran	1000	26	Unclear	15.62 ±2.5	Not reported	Cross-sectional	ELISA

Sample sizes ranged from 102 to 1000 subjects, with the number of participants infected with HCV ranging from 0 to 26. Infected individuals were all males, with one study unclear on the sex of the sample participants [18]. The mean ages ranged from 10.1 to 15.6 years, with age not reported in one study [16]. All studies utilised enzyme-linked immunosorbent assay (ELISA) to determine whether the children had HCV?

The methodological quality of the studies was appraised. Vahdani et al.’s [15], Ataei et al.’s [17] and Foroughi et al.’s [18] studies were considered of high quality whereas Fallah et al.’s [16] study was deemed to be of moderate quality.

**Fig. 2:**
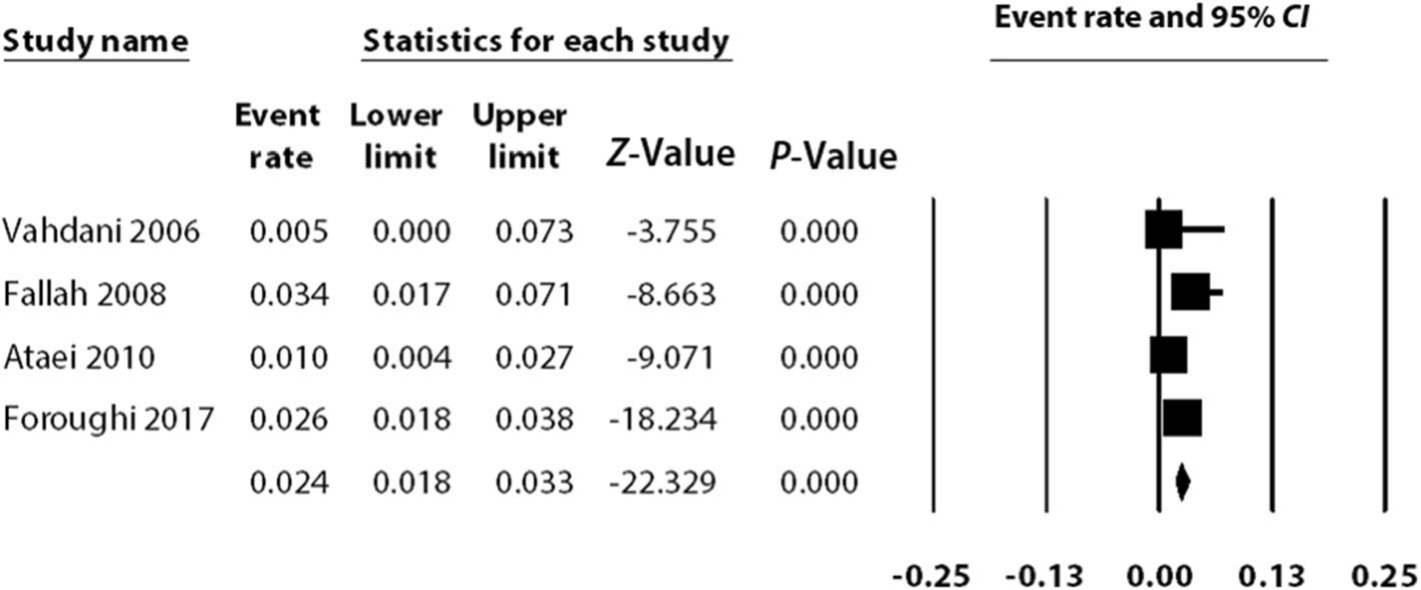
The overall prevalence of HCV among Iranian street children

Based on the fixed-effects model, the prevalence of HCV among street children in Iran was found to be 2.4% (95% *CI*: 1.8–3.3). The observed heterogeneity was 43.3%, which was found to be not statistically significant. Figure 2 is a forest plot of the included studies.

**Fig. 3:**
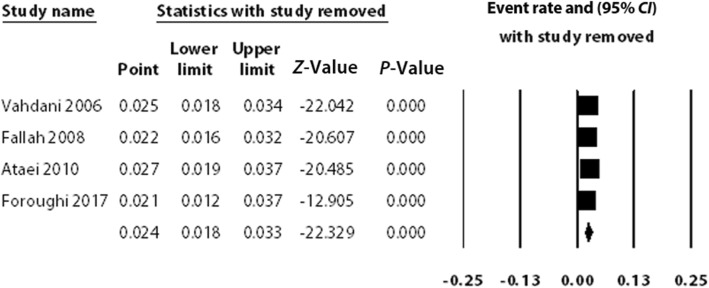
A sensitivity analysis

A sensitivity analysis showed that the results were stable. Before and after the sensitivity analysis, the results did not change, confirming the reliability of our findings, in that removing each included study had no impact (see Fig. 3).

The following risk factors were present in the children with HCV in the four selected studies: tattoos, cigarette smoking, drug addiction, alcohol abuse, sexual violence, having unprotected sexual intercourse and parents with a history of injection drug use. Unfortunately, there were not enough data to calculate the *OR*s.

## Discussion

The present study is a systematic and meta-analytical review of the prevalence of HCV among street children in Iran. As health ministry plans are developed in each country based on community needs, access to updated health statistics and solid scientific evidence is essential. Determining the frequency of the disease and high risk groups is very helpful [19]. As such, the present systematic review and meta-analysis has found a dearth of studies investigating the problem of HCV among street children in Iran.

According to the studies analysed in this review, the prevalence of HCV among street children in Iran was 2.4% as of 2017. This prevalence is higher in comparison with the general population in Iran, which was reported to be 0.6% in a meta-analysis study conducted in 2017 [20] or the 0.5% reported among blood donors conducted in 2013 [21]. This finding is a very serious warning for decision- and policy-makers in the Iranian health sector that street children are easily exposed to HCV and other infectious diseases. Unfortunately, as street children are at risk of various factors such as narcotics, tattooing, physical abuse, lack of proper health facilities, and lack of social and family support, they can be exposed to HCV easily [22]. In addition, the risk factors reported in the selected studies varied among the investigations and this could further explain this difference in prevalence.

The reviewed studies were carried out in two metropolitan areas: Tehran and Isfahan. Due to financial difficulties, many families migrate to larger cities to get away from social hardships such as addiction, divorce, neglect or abortion. Continuously wandering for hours along the streets exposes children to physical and psychological hazards, and, in some cases, to illness, such as blood-borne infectious diseases and STDs [23–25].

The prevalence of HCV among street children in Canada [26] was 12.6% in 2001, whereas in 1995 in Brazil [27] it was 3%: higher than the prevalence found in the reviewed studies. One of the most important reasons for a lower rate of HCV could be the implementation of the hepatitis B vaccination plan in Iran for all newborns [28], which could improve the health-literacy and the awareness about hepatitis, with studies confirming that this programme could significantly reduce the incidence of hepatitis in Iran [29–32]. Of course, social, cultural and health conditions can also affect the incidence of HCV [33, 34]. Paying attention to the prevalence of HCV among street children is important, and different countries should try to identify their prevalence rates in order to implement programs and policies [35–38].

### Limitations of study

This review has some limitations. Studies conducted on this topic are very limited and therefore researchers in Iran should research the spread of infections such as HCV among street children further. In addition, these studies should be conducted in different cities of Iran in order to provide a better understanding of the status of the infection based on geographical areas. Other limitations include a lack of information to calculate gender-based prevalence or risk factors that can have a significant impact on the disease. In addition, due to the small number of studies included, the publication bias and meta-regression to explore possible sources of heterogeneity could not be performed, even though heterogeneity was found to be less than 50% and, as such, not statistically significant.

Given the abovementioned limitations, further studies should be conducted among street children in Iran in order to improve the current knowledge of the problem and provide health decision- and policy-makers with better evidence for them to design social and economic support programmes.

## Conclusions

The findings of this study showed that the prevalence of HCV among street children in Iran is quite high, considerably higher in comparison with the general population of Iran or other clinical populations, such as blood donors. The health system should provide facilities for street children to be screened to quickly diagnose illnesses and prevent them from developing complications.

## Additional file

Additional file 1: Multilingual abstracts in the five official working languages of the United Nations (PDF 244 kb)

## Abbreviations

*CI*: Confidence interval
HCV: Hepatitis C virus
*OR*: Odds ratio
STD: Sexually transmitted disease
