# Supplementary material for: Prevalence of hepatitis C virus among street children in Iran
Source: Infect Dis Poverty. 2018 Oct 1;7:88. doi: 10.1186/s40249-018-0469-5 (PMC6166285; doi:10.1186/s40249-018-0469-5)
Supplement: Supplementary file 1 — Multilingual abstracts in the five official working languages of the United Nations (PDF 244 kb) [file 40249_2018_469_MOESM1_ESM.pdf]

Translation of the abstract into the five official working languages of the United Nations

انتشار فيروس الالتهاب الكبدي سي بين أطفال الشوارع في إيران

مسعود بهزادفار،حسن أبو الجاسم جورجي، عزيز ريزابور، ونيكولا لويجي براجازي

الملخص
الخلفية يُجبَر أطفال الشوارع على قضاء الكثير من الوقت بعيدا عن منازلهم ، وبعض هؤلاء الأطفال ليس لديهم بيوت على الإطلاق، نتيجة لمشاكل عائلية واقتصادية، والتي تجعلهم مُعرّضين للكثير من المشاكل الصحية، مثل فيروس الالتهاب الكبدي سي عدوى (HCV). فإيران، مثل العديد من البلدان الأخرى في العالم، تعاني من عبء أطفال الشوارع، إلا أن معدل فيروس الالتهاب الكبدي سي بين أطفال الشوارع غير معروف فعليا. وتهدف هذه الدراسة إلى تحديد نسبة انتشار فيروس الالتهاب الكبدي سي بين أطفال الشوارع في إيران.
الموضوع الرئيس أُجريت هذه المراجعة المنهجية والتحليل التلوي وفقا لإرشادات بنود التقارير المفضلة للمراجعات المنهجية والتحليلات التلوية. سُجّل بروتوكول هذه المراجعة في "بروسبيرو" تحت المصطلح التعريفي CRD42018082336 وقد أُجري بحث شامل للأعمال المطبوعة لتحديد الدراسات المنشورة التي أعدت تقارير عن انتشار فيروس الالتهاب الكبدي سي بين أطفال الشوارع في إيران. وقد أُجري البحث في العديد من قواعد البيانات العلمية الدولية والتي تشمل، شبكة العلوم، و PubMed®/MEDLINE®, Embase, Scopus, الباحث العلمي من جوجل و دليل دوريات الوصول الحر، فضلا عن قواعد بيانات إيرانية مثل MagIran و Barakathns. وأُختيرت الدراسات الصادرة بين عامي 1988 و ديسمبر 2017 ، والتي تحمل أي من الكلمات المفتاحية (شارع أو مشرد أو عمالة) و (أطفال أو طفل أو صغير) و (الالتهاب الكبدي سي أو فيروس التهاب الكبد سي أو اتش سي في أو التهاب الكبد أو الفيروس الكبدي) و كلمة إيران. بالأضافة على ذلك، أُجري بحث عن المنشورات غير المعلنة للوصول إلى الدراسات الأخرى التي يحتمل أن تكون ذات صلة. وأُجري البحث دون أية قيود لغوية. و تستقصي أربع دراسات إجمالي 1691 من أطفال الشوارع، أجريت بين عامي 2006 و 2017 تبيّن أنها صالحة للإدراج في المراجعة ولذلك حُلِلَت. وأُجريت ثلاث دراسات في طهران وواحدة في أصفهان. فاصل الثقة 1.8 - 3.3).وتبين أن معدل انتشار فيروس التهاب الكبد سي مرتفع بين أطفال الشوارع في إيران و يبلغ 2.4% (95%
الاستنتاجات: بما أن معدل انتشار فيروس الالتهاب الكبدي سي مرتفع جدا بين أطفال الشوارع في إيران، فينبغي على صانعي القرارات الصحية وصانعي السياسات إعطاء اهتمام أكبر لأطفال الشوارع وتوسيع قنوات الدعم الاجتماعية والاقتصادية. ويجب إجراء دراسات أكثر بين أطفال الشوارع في مختلف مدن إيران للإضافة إلى قاعدة المعرفة بفيروس الالتهاب الكبدي سي بين أطفال الشوارع في الدولة. وينبغي أن يوفر النظام الصحي تسهيلات لأطفال الشوارع ليتم فحصهم بهدف التشخيص السريع للأمراض ومنع حصول مضاعفات لهم.

Translated from English version into Arabic by Aseel Abdulrahman and Bashaier Allam, through

TRANSLATORS
WITHOUT BORDERS

伊朗街头儿童中丙肝病毒的流行情况

Masoud Behzadifar, Hasan Abolghasem Gorji, Aziz Rezapour and Nicola Luigi Bragazzi

摘要：
背景：由于经济和家庭问题，流浪儿童长期离家，其中一些人根本没有家，因而容易引起丙肝等众多健康问题。 同世界上许多其他国家一样，伊朗承有很多流浪儿童，然而其丙肝病

毒（HCV）的感染率却未被统计。本研究旨在明确伊朗流浪儿童的 HCV 感染率。
正文：本研究依据系统综述和 Meta 分析的优先报告条目进行系统综述和 Meta 分析。本研究方案在 PROSPERO 中注册号为 CRD42018082336。我们对对伊朗流浪儿童 HCV 流行率相关研究文献和报道进行了全面检索，七个数据库或搜索引擎包括 Web of Science、PubMed®/MEDLINE®、Embase，Scopus®、Google 学术和开放获取期刊目录等国际数据库，以及 MagIran 和 Barakathns 等伊朗数据库。本研究纳入了在 1988 年至 2017 年 12 月期间发表病包含以下任何一个关键词的文章:(街头、流浪、无家可归或劳力）和（儿童、幼儿或婴儿）和（丙型肝炎、丙型肝炎病毒、病毒性肝炎、肝炎或肝炎病毒）和伊朗。此外，我们检索了对灰色文献以获得其它可能相关的研究。此次检索是在没有限制语言。2006 年至 2017 年期间开展的四项调查共计 1691 名街头儿童被纳入本研究，其中三项在德黑兰，一项在伊斯法罕。研究发现伊朗街头儿童的 HCV 患病率很高，达 2.4％（95％CI：1.8–3.3）。
结论：在伊朗流浪儿童中，HCV 流行率很高。因此卫生决策者和政策制定者应该更加关注流浪儿童，扩大社会和经济支持渠道。也需对伊朗不同城市的流浪儿童进行进一步调查研究，以补充整个国家中流浪儿童的 HCV 患病情况。卫生系统应为流浪儿童提供筛查设施，以便快速诊断疾病并预防并发症。

Translated from English version into Chinese by Peng Song, edited by Jin Chen

Prévalence du virus de l'hépatite C chez les enfants des rues en Iran

Masoud Behzadifar, Hasan Abolghasem Gorji, Aziz Rezapour et Nicola Luigi Bragazzi

RÉSUMÉ

Contexte : Les enfants des rues sont souvent contraints de passer beaucoup de temps loin de chez eux et certains d'entre eux n'ont même pas de résidence du tout, dû à des problèmes économiques et familiaux, ce qui les rend vulnérables à de nombreux problèmes de santé tels qu'une infection du virus de l'hépatite C (VHC). L'Iran, tout comme beaucoup d'autres pays dans le monde, subit le fardeau des enfants des rues; cependant, le taux d'infection du VHC chez les enfants des rues demeure pratiquement inconnu. L'étude qui suit a tenté de déterminer la fréquence d'infections du VHC parmi les enfants des rues en Iran.
Corps principal : Un examen systématique a été conduit en suivant les lignes directrices de PRISMA (soit Points préférés pour les rapports de revues systématiques et de méta-analyses). Le protocole d'étude de cet examen a été enregistré dans la base de données PROSPERO sous le code d'identification CRD42018082336. Une étude documentaire exhaustive a été menée dans le but d'identifier les études publiées traitant de la fréquence des cas de VHC parmi les enfants des rues en Iran. Plusieurs bases de données universitaires internationales, incluant Web of Science, PubMed®/MEDLINE®, Embase, Scopus®, Google Scholar et le Directory of Open Access Journals (DOAJ), en plus des bases de données iraniennes telles que MagIran et Barakathns, ont été choisies pour les recherches. Les études publiées entre 1988 et 2017 présentant un ou plusieurs des termes suivants ont été choisies : (rue OU sans-abri OU travail) ET (enfants OU enfant OU nouveau-né) ET (hépatite C OU virus de l'hépatite C OU VHC OU hépatite virale OU hépatite OR hepacivirus)

ET Iran. De plus, une recherche de littérature grise a été menée dans le but d'obtenir d'autres études potentiellement pertinentes. Aucune restriction linguistique n'a été imposée dans le cadre de cette recherche. Quatre études, recensant un total de 1691 enfants des rues et menées entre 2006 et 2017, ont été jugées admissibles pour être inclues dans l'examen, et donc analysées. Trois études ont été menées à Téhéran et une à Isfahan. La fréquence de cas de VHC parmi les enfants des rues en Iran s'est révélée assez élevée, à 2,4 % (95 % *CI*: 1,8–3,3).
Conclusions : Puisque la fréquence des cas de VHC parmi les enfants des rues en Iran est plutôt élevée, les responsables des politiques et des mesures de santé devraient davantage porter attention aux enfants des rues et élargir leurs techniques de soutien social et économique. Il faudrait mener davantage d'études auprès des enfants des rues dans différentes villes d'Iran afin de renforcer les connaissances quant au VHC parmi les enfants des rues dans le pays. Le système de santé en place devrait fournir des services pour dépister les cas chez les enfants des rues afin de diagnostiquer rapidement les maladies et les aider à prévenir des complications de santé.

Translated from English version into French by Louis Gauvreau and Sophie N, through

TRANSLATORS
WITHOUT BORDERS

Заболеваемость вирусом гепатита С среди беспризорных детей в Иране

Масуд Бехзадифар, Хасан Аболгасем Горджи, Азиз Резапур и Никола Луиджи Брагацци

АННОТАЦИЯ

Краткая справка: Беспризорные дети вынуждены проводить много времени вдали от своих домов, а некоторые из них вообще не имеют дома из-за денежных и семейных проблем, вследствие чего они становятся беззащитными перед лицом множества заболеваний, таких как вирус гепатита С (ВГС). В Иране, как и во многих других странах мира, остро стоит проблема беспризорных детей. Однако при этом показатели заболеваемости ВГС среди них практически не изучены. Целью настоящего исследования является изучение заболеваемости вирусом гепатита С среди беспризорных детей в Иране.
Основная часть: Систематический обзор и метаанализ проводились в соответствии с рекомендациями, включёнными в «Предпочтительные параметры отчётности для систематических обзоров и метаанализа (PRISMA)». Протокол исследования данного обзора зарегистрирован в системе PROSPERO под идентификационным номером CRD42018082336. С целью выявления опубликованных исследований на тему заболеваемости вирусом гепатита С среди беспризорных детей в Иране был проведён всесторонний анализ литературы. В ходе исследования была использована информация из баз данных научных публикаций, включая платформы Web of Science, PubMed®/MEDLINE®, Embase, Scopus®, Google Scholar и Directory of Open Access Journals, а также иранские базы данных, такие как MagIran и Barakathns. Была сделана выборка исследований, опубликованных в период между 1988 годом и декабрем 2017 года по следующим ключевым словам: (беспризорные ИЛИ

бездомные ИЛИ труд) И (дети ИЛИ ребёнок ИЛИ младенец) И (гепатит С ИЛИ вирус гепатита С ИЛИ ВГС ИЛИ вирусный гепатит ИЛИ hepacivirus) И Иран. Кроме того, был проведен обзор литературы, не индексированной в медицинских базах данных, для выявления прочих исследований, потенциально относящихся к данному вопросу. Поиск исследований выполнялся без каких-либо языковых ограничений. Четыре проведенных в период с 2006 года по 2017 год исследования, предметом которых являлось изучение состояния 1 691 беспризорных детей, были признаны удовлетворяющими требованиям для включения в обзор и, соответственно, проанализированы. Были проведены три исследования в Тегеране и одно в Исфахане. Было выяснено, что процент заболеваемости ВГС среди беспризорных детей в Иране высок и достигает 2,4% (95% *CI*: 1,8–3,3).

Выводы: Так как уровень заболеваемости ВГС среди беспризорных детей в Иране довольно высок, органы, ответственные за принятие решений, а также за формирование и реализацию политики в области здравоохранения, должны уделять больше внимания беспризорным детям и развивать каналы их поддержки, как социальной, так и экономической. Представляется необходимым проведение дальнейших исследований среди беспризорных детей в различных городах Ирана, чтобы расширить базу знаний о заболеваемости вирусом гепатита С среди беспризорных детей в стране. Система здравоохранения обязана обеспечить инфраструктуру для диспансеризации беспризорных детей с целью своевременной диагностики заболеваний и предотвращения развития их осложнений.

Translated from English version into Russian by Galina Dmitrieva and Liudmila Tomanek, through

TRANSLATORS
WITHOUT BORDERS

Prevalencia del virus de la hepatitis C en niños de la calle en Irán

Masoud Behzadifar, Hasan Abolghasem Gorji, Aziz Rezapour y Nicola Luigi Bragazzi

RESUMEN

Antecedentes: Los niños de la calle se ven obligados a pasar mucho tiempo fuera de casa y algunos no tienen hogar debido a problemas económicos y familiares, por lo que son vulnerables a numerosos problemas de salud, como la infección por el virus de la hepatitis C (VHC). Muchos países tienen este problema como Irán, pero en ellos la tasa de VHC en niños de la calle es prácticamente desconocido. El objetivo de este estudio fue determinar la prevalencia del VHC en niños de la calle en Irán.

Cuerpo principal: Se realizó una revisión sistemática y un metaanálisis según las pautas de Preferred Reporting Items for Systematic Reviews and Meta-Analyses. El protocolo de estudio de esta revisión se registró en PROSPERO con el código de identificación CRD42018082336. Se llevó a cabo una búsqueda exhaustiva de estudios publicados sobre la prevalencia del VHC en niños de la calle en Irán. Se utilizaron varias bases de datos académicos internacionales, entre ellas: Web of Science, PubMed®/MEDLINE®, Embase, Scopus®, Google Académico y el Directorio de Open

Access Journals, además de las bases de datos iraníes como MagIran y Barakathns. Se seleccionaron estudios publicados entre 1988 y diciembre de 2017 que incluyeran cualquiera de las siguientes palabras clave: "calle"O "personas sin hogar" O "trabajo"; Y "niños" O "niño" O "infante"; Y "hepatitis C", "virus de hepatítis C" O"hepatítis viral" O "hepatitis" O "hepacivirus"; E "Irán". Además, se buscó "literatura gris" con el objeto de acceder a otros estudios potencialmente relevantes. La búsqueda se realizó sin restricción de idioma. Cuatro estudios realizados entre 2006 y 2017, en los que se entrevistó a un total de 1691 niños de la calle, se consideraron adecuados para incluirlos en la revisión y, por tanto, para su análisis. Se realizaron tres estudios en Teherán y uno en Isfahan. La prevalencia del VHC entre los niños de la calle en Irán resultó ser alta, del 2.4% (95% *CI*: 1.8–3.3).

Conclusiones: Puesto que la prevalencia del VHC entre los niños de la calle en Irán es bastante alta, los responsables de la toma de decisiones y de las políticas de salud deberían prestar más atención a estos niños y ampliar los canales de apoyo, tanto sociales como económicos. Se deberán realizar más estudios entre los niños de la calle en diferentes ciudades de Irán para agregarlos a la base de conocimientos sobre el VHC entre los niños de la calle en este país. El sistema de salud debería contar con recursos para examinar a los niños de la calle a fin de diagnosticar rápidamente las enfermedades y evitar que desarrollen complicaciones.

Translated from English version into Spanish by Sara Solá Portillo and Guadalupe Barua, through

TRANSLATORS
WITHOUT BORDERS
